# American Medical Association

**Published:** 1891-09

**Authors:** 


### Reports of Society Meetings.

### AMERICAN MEDICAL ASSOCIATION.

*Wednesday, May 6, 1891.—Morning Session.*

### SECTION OF ORAL AND DENTAL SURGERY.

(Continued from page 574.)

*Dr. Talbot.*—We will now hear Dr. Andrews's paper on "Growth of the Cementum."

(For Dr. Andrews's paper, see page 587.)

*Dr. Marshall.*—Mr. President, as I feel I shall be obliged to absent myself, I want to make a report from the nominating committee: for Chairman of the Section, Dr. Taft, of Cincinnati, and for Secretary, Dr. E. S. Talbot, of Chicago.

*Dr. Taft.*—I would rather that some one else would accept; I do not absolutely decline, but think some one else can hold the position better.

Referred back to the committee to report later.

*Dr. Talbot.*—We will now proceed with the discussion of Dr. Andrews's paper.

*Dr. Taft.*—I want to make a remark in reference to this matter. Dr. Andrews referred to what is ordinarily denominated interglobular spaces and gives the location as between the enamel and dentine.

*Dr. Andrews.*—Not between the enamel and dentine, just within the dentine.

*Dr. Taft.*—I know; I understood you to say this position has been taken by others, that they located the interglobular spaces in the granular layer; but that is not the location nor the appearance as originally described by Czermack, as recorded by Kölliker. If the structure denominated intergolubar spaces is to be found in the granular layer, then what is this globular structure within the dentine?

*Dr. Andrews.*—The one is like the other, with this difference, the calcified globules near the cementum are small, while in the dentine of the crown they are much larger.

*Dr. Taft.*—We have not ordinarily applied the term interglobular space to this layer between the enamel and dentine. In some instances there is a very large mass of it of considerable thickness. Another peculiarity of that layer is, it seems to consist, whether thick or thin, of mere little globular granules. In some instances there seems to be tubuli, or something of the kind, extending through it to a very considerable extent. I wish I knew more about that than I do, and about the interglobular spaces.

*Dr. Andrews.*—Interglobular spaces are found with the globules in layers, the layers of formation, some near the enamel, others near the pulp. In the teeth of some fishes the whole matrix is globular. In the human tooth it is an evidence of lack of nourishment while the tooth is forming. The globules do not fuse, they are arrested and calcified as globules, not forming a perfect layer of matrix. The material between the globules is not calcified substance. Some teeth have very little, others no evidence of a granular layer, so called, others have a good deal. Some have hardly a trace of what we call interglobular spaces, in the dentine of the crown, others will have a large amount. It is an arrest in the developmental process in the formation of the tooth while it is forming a layer of matrix. For some reason the globules have become calcified while in the globular form, before they fused together. The tissue of the interglobular space is not fully calcified, while the globules always are.

*Dr. Allan.*—This theory of Dr. Andrews, in which I believe he does not stand alone, is one that I think we should place in the status that the old Scotch law allowed: when a person was tried and was neither innocent nor guilty, it was "not proven." The three structures of which the two substances are composed—enamel, dentine, and cementum—are as diverse in their character and structure as any three tissues well can be. According to this theory of Dr. Andrews, all three of these substances, materials, or tissues—that is, the matrix of them—have a like developmental basis.

*Dr. Andrews.*—The lime base of the matrix material, the basis substance, is the same in all. It is the calcified tissue.

*Dr. Allan.*—All three are formed from cells, which have special duties required of them. Enamel is only two to four per cent. animal basis, ninety-six to ninety-eight per cent. mineral. Dentine again is thirty to forty per cent. animal, the balance mineral. I have examined a great many of these specimens of Dr. Andrews's, and I have endeavored to see exactly as he sees, and I think I do see what he sees; but I don't interpret it as he does, for this reason: The material, whatever it is, the calcoglobulin, is thrown out in the shape of little globules, which vary greatly in size and character. The position may or may not be a vital condition. All these preparations of Dr. Andrews were made with the greatest care and from sections as little changed in preparation as possible. The calcification was produced by a very weak solution of acid, and every care taken to change the tissue as little as possible; but—and I hope I may be excused in saying so—it is not proven that these are not post-mortem appearances; and a long series of experiments will have to be carried on to prove whether they are post-mortem or living appearances. You do have unmistakable indications of these globular layers; and they are beautifully shown in these preparations. These appearances are not constant; now you see them, and again you don't.

*Dr. Andrews.*—I have been speaking in all of the matrix substances, not its animal base: that is another matter. The globules are always present in my specimens.

*Dr. Allan.*—But the main point I wish to show is that I do not see why this cannot be in a measure post-mortem appearance. I do not see why the fluids that have been used in preparing the tissue, glycerin, in which they are mounted, may not have some cause in giving them their characteristic appearance. It is a most interesting point. Whether the two kinds of cells that go to make up the formation of dentine really exist or do not exist, one the fibre-forming and the other the matrix-forming. We often see appearances that would indicate double or dual nature of the cells that form dentine. In enamel it is not so. We do not often treat and we seldom see a dual character of cells. To be sure, the enamel is so totally different that the one form could be dispensed with; but in the cementum again it could not be. There we have a large mass of animal matter, and we have a composite structure.

Now, so far as these interglobular spaces are concerned, Dr. Andrews's specimens were taken under high power. The interglobular spaces in dentine are vastly different in size from those interglobular spaces that Dr. Andrews threw on the screen. Take a tooth and make a section of it, and you will find interglobular spaces sometimes; I should say they would extend across nearly one-tenth of the thickness of the tooth. These globules are much smaller than the average size of the interglobular spaces you find in dentine.

Now, I am not saying that this theory is not true. I believe that the evidence Dr. Andrews offers is very strong and confirmatory evidence. I only say there are still some points open for investigation before we can say proven and adopted. I don't know of any one in the profession who deserves greater respect than does Dr. Andrews, or who has done more disinterested work; and the results have been certainly most gratifying. I can say this heartily and truthfully; and I know of no one who has given his specimens more freely and generally to others for the purpose of examining them.

*Dr. Taft.*—I wish more attention were given to the discrimination of these two conditions. The large spaces within the dentine that are ordinarily denominated globular spaces are very different in size from any condition that is found in the granular layer. In the granular layer these little spheres are granulous and thoroughly calcified. This peculiar abnormal structure in dentine, marked by these lines in a greater or less degree, is defective; and then these circles or segments of circles within dentine are not in a normal condition: occasionally you find deep lines running through, and in some of them no organization of that kind at all.

The globules are thoroughly calcified. The interglobular space is not perfect, but the globular spaces are perfect. The tissue between the globules is not calcified, but the space between the globules is soft. I assume this matter is clear to Dr. Andrews, but I wanted him to make it clear to me and to all of us.

*Dr. Andrews.*—If you will examine sections of teeth where there are interglobular spaces, you will find the globules are calcified. In dried sections, the spaces between the globules will show dark under the microscope in the same way that the tubes do. It is because the spaces are not calcified, and when dry they fill with air. Some of the globules are only slightly attached to the matrix, while others show only a portion of their contour.

*Dr. Allan.*—Do I understand you to say that this is a secretion?

*Dr. Andrews.*—It is supposed that the lime in the form of minute calcospherites comes from the blood. These pass through the cells, and are seen to be merging together forming large globules, or mulberry-shaped masses, and these fusing form the perfect layer of matrix, if there is no arrest of development while they are in this stage.

*Dr. Taft.*—You make the point, it was arrested in development?

*Dr. Andrews.*—Yes, when interglobular spaces are found.

*Dr. Taft.*—You mean that the environments are not perfect?

*Dr. Andrews.*—Yes, sir.

The President called on Dr. Grady to read his paper on "The Use and Abuse of Dental Charity."

(For Dr. Grady's paper, see page 592.)

The paper was discussed by Dr. Taft, of Cincinnati, Dr. Clara McNaughten, of Washington, Dr. J. L. Williams and Dr. R. R. Andrews, of Boston, and Dr. E. S. Talbot, of Chicago, after which the following resolutior was unanimously adopted:

*Resolved*, That this section would suggest to the National Association of Dental Faculties the importance of some action towards correcting the abuses of the charities of infirmaries of dental schools.

(To be continued.)

